# m^6^A Reader YTHDC2 Promotes Radiotherapy Resistance of Nasopharyngeal Carcinoma via Activating IGF1R/AKT/S6 Signaling Axis

**DOI:** 10.3389/fonc.2020.01166

**Published:** 2020-07-31

**Authors:** Jun-Ju He, Zhi Li, Zhuo-Xian Rong, Jie Gao, Yun Mu, Yi-Di Guan, Xin-Xin Ren, Yu-Yuan Zi, Li-Yu Liu, Qi Fan, Ming Zhou, Yu-Mei Duan, Qin Zhou, Yue-Zhen Deng, Lun-Quan Sun

**Affiliations:** ^1^Xiangya Cancer Center, Xiangya Hospital, Central South University, Changsha, China; ^2^Key Laboratory of Molecular Radiation Oncology Hunan Province, Changsha, China; ^3^Cancer Research Institute and School of Basic Medical Sciences, Central South University, Changsha, China; ^4^Department of Oncology, Xiangya Hospital, Central South University, Changsha, China; ^5^Hunan International Science and Technology Collaboration Base of Precision Medicine for Cancer, Changsha, China; ^6^National Clinical Research Center for Gerontology, Changsha, China

**Keywords:** m^6^A, YTHDC2, nasopharyngeal carcinoma, IGF1R, PI3K-AKT signaling

## Abstract

N6-methyladenosine (m^6^A) modification has been reported as a critical regulator of gene transcript expression. Although m^6^A modification plays important roles in tumor development, its role in therapeutic resistance remains unknown. In this study, we aimed to examine the expression level of m^6^A-modification related proteins and elucidate the effect of m^6^A-related proteins on radiation response in nasopharyngeal carcinoma (NPC). Among the genes that participated in m^6^A modification, YTHDC2, a m^6^A reader, was found to be consistently highly expressed in radioresistant NPC cells. Knocking down of YTHDC2 expression in radioresistant NPC cells improved the therapeutic effect of radiotherapy *in vitro* and *in vivo*, whereas overexpression of YTHDC2 in radiosensitive NPC cells exerted an opposite effect. Bioinformatics and mechanistic studies revealed that YTHDC2 could physically bound to insulin-like growth factor 1 receptor (IGF1R) messenger RNA and promoted translation initiation of IGF1R mRNA, which in turn activated the IGF1R-AKT/S6 signaling pathway. Thus, the present study suggests that YTHDC2 promotes radiotherapy resistance of NPC cells by activating the IGF1R/ATK/S6 signaling axis and may serve as a potential therapeutic target in radiosensitization of NPC cells.

## Introduction

Nasopharyngeal carcinoma (NPC) is a malignant tumor arising from the epithelium of the nasopharynx, and most cases of NPC usually occurred in Southeast Asia and Southern China (1–3). NPC is strongly associated with the Epstein-Barr virus infection, heredity, and environmental factors. At present, radiotherapy (RT) is the preferred treatment for patients with NPC (4–6). However, some cancer cells of NPC are insensitive to RT, which has resulted in therapeutic failure and recurrence. Therefore, the molecular mechanism underlying radiation resistance should be thoroughly studied, with the aim to identify promising targets to alleviate radiotherapy resistance of patients with NPC.

N^6^-methyladenosine (m^6^A) refers to methylation of the adenosine base at the nitrogen-6 position. It is the most abundant modification in messenger RNA (7) and is also found in ribosomal RNA, transfer RNA, and small nuclear RNA as well as some non-coding RNA (8, 9). The m^6^A RNA modification is a dynamic and reversible process coordinated by methyltransferase complex (m^6^A “writer”), demethylases (m^6^A “eraser”), and m^6^A-binding proteins (m^6^A “reader”) (10, 11). The m^6^A writer is a multicomponent complex consisting of METTL3, METTL14, WTAP, KIAA1429, RBM15, RBM15B, and ZC3H13. The catalytic function is performed by METTL3, while METTL14 serves as an RNA-binding platform (11). FTO and ALKBH5 were identified as RNA demethylases removing m^6^A modification in RNA (12, 13). To date, several m^6^A reader proteins reported in the mammalian genome directly recognized the m^6^A site and influenced RNA function by modulating RNA stability, splicing, translocation, translation, microRNA processing, circRNA ring formation, RNA-protein interaction, and so on (11). Recent studies demonstrated that abnormal expression of m^6^A modification proteins promotes cancer cell growth, survival, and tumor initiation and progression. For instance, an unexpected high expression of FTO in certain subtypes of acute myeloid leukemia promoted leukemogenesis and inhibited leukemia cell differentiation by regulating the m^6^A level in mRNA of ASB2 and RARA (14). Furthermore, ALKBH5 induced by hypoxia promoted stem cell self-renewal in breast cancer by mediating NANOG mRNA methylation (15). With respect to m^6^A writers, one study showed that knockdown of METTL3 or METTL14 facilitates stemness and tumorigenesis of glioblastoma stem cells (16). In contrast, another report demonstrated that high expression of METTL3 in human glioblastoma tissues was a predictor of poor prognosis (17). A similar controversy with respect to m^6^A writer has been observed in some studies regarding hepatocellular carcinoma (18, 19). For m^6^A readers, the m^6^A-binding proteins have been suggested to play some important roles in tumorigenesis and tumor progression. For example, YTHDF2 facilitated c-MYC and CEBPA mRNA degradation by binding to the m^6^A site in 5'UTR and coding region of c-MYC and CEBPA mRNA, resulting in leukemia cell proliferation (20). Although m^6^A modification proteins are involved in multiple processes of tumorigenesis and development, few studies have explored the effect of m^6^A on therapeutic responses of cancer.

YTH domain containing 2 (YTHDC2) is a member of DExD/H box RNA helicase family proteins, containing ATP binding motifs and RNA binding motifs. YTHDC2 regulates mRNA translation and stability by recognizing m^6^A modification. Previous studies demonstrated YTHDC2 as an essential regulator involved in the switch from mitosis to meiosis during spermatogenesis (21–23). Moreover, Tanabe et al. found that knockdown of YTHDC2 inhibited the expression of several proteins involved in the metastasis of colon cancer (24). A recent study indicated that the elongation-promoting biological process of CDS methylation required the RNA helicase-containing m^6^A reader YTHDC2 (25). In the present study, YTHDC2 was found to be highly expressed in radioresistant NPC cells. Knocking down of YTHDC2 in radioresistant NPC cells improved the therapeutic effect of radiotherapy *in vitro* and *in vivo*, whereas overexpression of YTHDC2 in radiosensitive NPC cells exerted an opposite effect. Bioinformatics and mechanism studies revealed that YTHDC2 could physically bind to insulin-like growth factor 1 receptor (IGF1R) messenger RNA and promote translation initiation of IGF1R mRNA, which in turn activated the IGF1R-AKT/S6 signaling pathway. Thus, the present study suggests that YTHDC2 promotes radiotherapy resistance of NPC cells by activating the IGF1R/ATK/S6 signaling axis.

In summary, our study demonstrates that the m^6^A reader YTHDC2 promotes radioresistance of nasopharyngeal carcinoma via a translation-dependent pathway.

## Materials and Methods

### Patients, Tissue Specimens, and Follow-Up

A total of 105 paraffin-embedded primary nasopharyngeal carcinoma (NPC) samples were obtained from Xiangya Hospital. A total of 105 NPC cases were recruited between January 2017 and July 2019. NPC samples were collected with complete pathological and clinical data. The follow-up period for assessing radiotherapy efficacy was defined as the first time NPC patients were treated with radiation to half year after radiation course. All samples were deidentified and all patients signed informed consents. The study was approved by the Institute Research Medical Ethics Committee of Central South University.

#### Patient Inclusion Criteria

The inclusion criteria of 105 cases included in our study were as follows: (1) pathologically proven squamous cell carcinoma (SCC) of locally advanced nasopharyngeal carcinoma (AJCC 7th staging system III–IVa); (2) the existence of tissue blocks available for our research; (3) first line IC and cisplatin-based concurrent chemotherapy regimen; (4) the MRI imagine evaluation data after radiochemotherapy are available, and the response is partial response or complete response; (5) without anticancer treatment prior to primary RT, or surgery after RT; (6) adequate organ function. This retrospective study was approved by the Clinical Research Ethics Committee of the Xiangya Hospital of Central South University.

#### Treatment and Evaluation

All patients received three cycles or two cycles of the IC regimen: TP (consisting of docetaxel [1 day of 75 mg/m^2^] and cisplatin [1 day of 75 mg/m^2^), 3 weeks per cycle. All patients were treated with external beam intensity modulated radiation therapy (IMRT), concurrent with 2 or 3 cycles of cisplatin [80–100 mg/m^2^/cycle, at least 200 mg/m^2^]. The target volumes were defined with reference to International Commission on Radiation Units and Measurements (ICRU) reports No. 50 and No. 62. A total dose of 70.4 Gy in 32 fractions to the PGTVnx (GTVnx +3 mm margin), 66 Gy to 70.4 Gy in 32 fractions to the PGTVnd, 60.8 Gy in 32 fractions to the PTV1, and 54Gy in 30 fractions to the PTV2 were prescribed. All patients were treated with one fraction daily, 5 days per week. Dose limits for the critical tissue structures and plan evaluation were as defined by the Radiation Therapy Oncology Group (RTOG) 0225 (26). The patients were re-examined by MRI and evaluate the efficiency 3 months after they finished the concurrent radiochemotherapy. The tumor response in different patients was evaluated by two independent clinicians on the basis of evaluation criteria for efficacy of solid tumors (RECIST) (27), which classified patients into CR, PR, SD, and PD according to the response of primary and nodal disease. According to the evaluation criteria, we selected 63 cases of CR and 42 cases of PR in our study.

### Cell Lines and Cell Culture

CNE2 was established from a 68-year-old male patient with nasopharyngeal carcinoma (28) and CNE2-IRR (CNE2-ionizing radiation radioresistent cell line) was derived from CNE2 after a prolonged exposure of irradiation (29). HK1, a generous gift from Prof. Ya Cao (Cancer Research Institute, Central South University), was established from a recurrent nasopharynx carcinoma of a Chinese 17-year-old male patient (30), and HK1-IRR (HK1-ionizing radiation radioresistent cell line) was derived from HK1 after a prolonged exposure of irradiation. CNE2, CNE2-IRR, HK1, and HK1-IRR were cultured in RPMI-1640 medium supplemented with 10% fetal bovine serum (FBS). All cell lines were maintained at 37°C with 5% CO_2_. All cell lines accepted cell line authentication service.

### Plasmid Construction and Transfection

Human wildtype YTHDC2 was generated using the following primers: forward: 5′-ATGTCCAGGCCGAGCAGCGTCTCCCCGCGG-3′ and reverse: 5′-TCAATCAGTTGTGTTTTTTTCTCCCAAGGG-3′, and then subsequently cloned into lentivector-based pLVX-IRES-Puro (Addgene). ShRNA targeting YTHDC2 was designed using software provided by Qiagen (Valencia, CA, USA) and cloned into the pLKO.1-TRC vector. The sequences of shRNA are as follows:

ShYTHDC2_1#(pLKO.1): 5′-CCGGCGGAAGCTAAATCGAGCCTTTCTCGAGAAAGGCTCGATTTAGCTTCCGTTTTTTG-3′

ShYTHDC2_3#(pLKO.1): 5′-CCGGGCCTTGGATGTAAATCTCTTTCTCGAGAAAGAGATTTACATCCAAGGCTTTTTTG-3'.

### Lentivirus Production, Precipitation, and Transduction

Lentivirus for shRNA-scramble, shRNA-YTHDC2_1#, shRNA-YTHDC2_3#, pLVX-Vec, pLVX-YTHDC2 were packaged with pMD2.G and psPAX2 (purchased by addgene). Briefly, 2.24 μg psPAX2, 0.76 μg pMD2.G and 1.5 μg pLKO-shYTHDC2 were co-transfected into HEK-293T cells in 60 mm dish with ViaFect™ Transfection Reagent (Promega). The Lentivirus particles were harvested at 48 and 72 h after transfection and concentrated with PEG8000 Virus Precipitation Solution (the concentrations of PEG-8000 and NaCl in the stock solution are 40% (W/V) and 1.2M, respectively). Finally, the concentrated lentivirus particles were directly added into the NPC cells and incubated at 37°C for 48 hr before they were washed by PBS. One μg/ml puromycin (A1113803, Invitrogen) was added into culture medium for the selection of stable NPC cell lines.

### Western Blot

NPC cell lines were lysed in a standard RIPA buffer containing proteinase and phosphatase inhibitors (Roche, Indianapolis, IN, USA), and the protein concentrations were quantified using Bradford reagent (Bio-Rad). Then prepared protein was separated via SDS-PAGE and transferred to NC membrane (Pierce Biotechnologies Inc., Rockford, IL, USA). The primary antibodies used in the study include anti-METTL3 (15073-1-AP, Proteintech), METTL14 (HPA038002, Sigma), FTO(ab92821, Abcam), ALKBH5 (HPA007196, Sigma), WTAP (sc-374280, Santa Curz), YTHDF1 (17479-1-AP, Proteintech), YTHDF2 (24744-1-AP, Proteintech), YTHDF3 (ab103328, Abcam), YTHDC1 (GTX32976, GeneTex), YTHDC2 (HPA037364, Sigma), IGF1R (3027,CST), AKT (9272, CST), Phospho-AKT (ser473, CST), S6 Ribosomal protein (2217, CST), and Phospho-S6 Ribosomal protein (ser240/244)(2215, CST). The bound antibodies were detected with HRP-conjugated secondary antibodies (Abcam). Densitometric analysis of WB results was performed with ImageJ software.

### Cell Viability and Apoptosis Array

Cell viability was detected by CCK-8 (Biomake) following the manufacturer's guideline. All viability experiments were repeated in three independent times, and Student's *t*-test was used to calculate statistical significance. Cell apoptosis assay was carried out according to the apoptosis kit (BD biosciences).

### RNA Extraction and Quantitative RT-PCR Analysis

Total RNA was isolated using TRIzol (Invitrogen). A total of 1,000 ng RNA was reverse-transcribed into cDNA in a total reaction volume of 20 μl with PrimeScriptTM RT Reagent Kit according to manufacturer's instructions (Takara). The qPCR primer sequences were listed in Supplementary Table 1. Real-time PCR was performed using SYBR premix Taq via CFX96 Real-Time PCR Detection System (Bio-Rad, Richmond, CA, USA). The 2^−ΔΔCT^ method was used to calculate the relative expression of YTHDC2. The ΔΔCt (ddCt) method was as described below:

ΔCt = Ct (target gene)-Ct (ref gene) and
ΔΔCt = ΔCt (target sample)-ΔCt (ref sample).

We got the relative mRNA expression between two samples (parental cells and radioresistant cells) by calculation of the following formula:

Relative mRNA expression = B/A.
A (Parental cells): 2^−ΔΔCT^.
B (radioresistant cells):2^−ΔΔCT^.

### Immunohistochemistry

Paraffin-embedded NPC samples were collected from Xiangya Hospital of Central South University. Tissue sections were deparaffinized, rehydrated, and subjected to antigen retrieval, and endogenous peroxidases were blocked. The sections were washed three times with 0.01 mol/L PBS (2 mmol/L NaH_2_PO_4_, 8 mmol/L Na_2_HPO_4_, and 150 mmol/L NaCl). Then, 0.01 mol/L PBS supplemented with 5% normal goat serum and 0.1% Triton X-100 was used to block the sections, followed by an incubation with anti-YTHDC2 (1:1,000; Sigma, HPA037364) overnight at 4°C. After three times washing in PBS and treatment with adjuvant for 20 min at 37°C, the sections were exposed to secondary antibody for 2 h at 37°C. The immunohistochemical reaction was visualized with 3, 3, 0-diaminobenzidine (DAB) for 3 min. All sections were counterstained with haematoxylin. Both the staining intensity and extent of protein expression were automatically scored by Vectra 2 system (Perkin-Elmer, USA).

### Colony Formation Assay

NPC cells were seeded in 6-well plates at densities of 1 × 10^3^ per well. Cells were exposed to radiation 0, 2, 4, 6, and 8 Gy at first day. Twelve days later, cells were washed three times with cold PBS and then stained with 0.2% crystal violet (containing methanol). Colonies consisting of more than 50 cells were defined as surviving colonies. All viability measurements are normalized with the 0 Gy group. Cell survival curves were fitted by Graph Pad software.

### Soft Agar Colony Forming Assay

Two thousand cells/well were plated in 24-well, flat-bottomed plates using a two-layer soft agar system in a volume of 400 μl/well. After the incubation for 15 days, colonies were counted and measured. Four fields were randomly selected for colony counts (>50 μm diameter and >100 μm diameter) under an inverted microscope (Leica DMI4000B, Germany) at 40× magnification. The freeware ImageJ (National Institutes of Health, Bethesda, MD, USA) and Adobe Photoshop (Adobe Systems, San Jose, CA, USA) were used in colony diameter measurement. Thirteen colonies were randomly selected for diameter measurement. Colony volume was calculated using the formula: volume = (length × width^2^)/2.

### RNA Stability Assays

CNE2-IRR cells with or without YTHDC2 knockdown were treated with actinomycin D at final concentration of 5 μg/ml for 0, 2, and 4 h before collected. Total RNA was isolated using TRIzol (Invitrogen) and analyzed by RT-PCR. The turnover rate was assessed according to a previously published paper (14).

### Polysome Profiling

We followed the reported protocols with the following modifications (31, 32). CNE2-IRR cells were transduced with shCON, shYTHDC2_1#, and shYTHDC2_3# lentivirus and selected with puromycin (1 μg/ml). Before collection, cycloheximide (CHX) was added to the culture media at 100 mg/mL for 5 min. Fifty million cells from each group were harvested, rinsed in cold PBS with 100 mg/mL CHX, and quickly frozen in dry ice before lysis. The lysis buffer was formulated as 20 mM HEPES (pH7.6), One hundred mM KCl, 5 mM MgCl_2_, 100 mg/ml CHX, 1% Triton X-100, with freshly added 1:100 protease inhibitor (Roche) and 40 U/ml SUPERasin (Ambion). The sample was then fractionated into 24 fractions (0.5 mL per fraction) and analyzed by Gradient Station (BioCamp) equipped with ECONOUV monitor (BioRad, Hercules, CA) and Gilson FC203B fraction collector (Mandel Scientific, Guelph, Canada). RNA was purified from whole cell, 40S, 60S, 80S, and Polysome fractions and subjected to qPCR analysis. Expression of IGF1R in each fraction was normalized to TUBULIN as well as Input.

### RNA Immunoprecipitation

We following the previous protocols with the following modifications (33, 34). CNE2-IRR cells were infected with Flag and Flag-YTHDC2 lentivirus-particles and puromycin (1 μg/ml) was added to culture medium for selection. Prior to lysing the cells, cells (1 × 10^7^) were UV–cross-linked at 254 nm (2,000 J/m^2^). After three washing with cold PBS, the cells were scraped with 1 ml ice-cold PBS and collected in 1.5 ml EP tube. Centrifuge cells at 300 g for 5 min at 4°C, then remove the supernatant and resuspend cells in 200 μl of lysis solution (10 mM HEPES [pH 7.0], 100 mM KCl, 5 mM MgCl_2_, 25 mM EDTA, 0.5% Nonidet P-40, 1% Triton X-100, 0.1% SDS, 10% glycerol, 1 mM DTT, 80 U/mL of RNase inhibitor RiboLock, a cocktail of proteinase inhibitors, PIC) for 30 min vigorous shaking. After centrifuging the 1.5 ml tube, we transferred supernatant to a new 1.5 ml tube and added DNA I for 30 min. Next, whole cell lysate was incubated with anti-flag antibody for 4 h at 4°C with continuous mixing (rotating or roller mixer). Forty microliters of protein A/G agarose beads were added to recruit RNA–protein complexes. RNAs associated with PTBP1 were recovered with Trizol-chloroform and analyzed by RT-PCR or qPCR.

### NPC Xenograft Model

All animal procedures were approved by the Animal Ethics Committee of Central South University; 2 × 10^6^ cells resuspended in 50 μl of Matrigel (Corning) were subcutaneously injected into 4–6 weeks old male nude mice. When tumor volumes reached 150–200 mm^3^, animals were divided into control group and radiotherapy group. In the radiotherapy group, tumors were treated with a single irradiation (4 Gy) when tumor volumes reached approximately 150–200 mm^3^. The tumor stopped growing in the next few days and then restarted growth. Three weeks later, the tumors were harvested, and tumor weight and volume were measured. Tumor volumes were calculated by l × w^2^/2 (l: tumor length, w: tumor width).

### Microarray-Based RNA Expression Profiling, Survival Analysis, and Correlation Analysis

The raw data of GSE48501 (35) was downloaded from a Gene expression Omnibus (GEO) database for exploring the different expressions of mRNA between radioresistant NPC cells and radiosensitive NPC cells. Next, the heatmap based on 12 differentially expressed m6A modification genes in each chip data were plotted using the heatmap package of the R software. The NPC dataset of GSE102349 (36) was used to perform Recurrence-free survival rate (RFS) and correlation analysis.

### DNA Preparation and Methylation-Specific PCR

The nasopharyngeal carcinoma cells were digested with trypsin-EDTA before collection, and genomic DNA was then prepared by using the proteinase K method. DNA was dissolved in TE buffer and stored at −20°C. Bisulfite modification of genomic DNA and methylation-specific PCR analysis were performed according to the protocol of the manufacturer (TIANGEN). The used primers are listed in Supplementary Table 3.

### Statistical Analysis

Statistical analyses were performed using SPSS 20.0 (SPSS) and GraphPad Prism, version 7. Flow cytometry data was analyzed using FlowJo 10.01. The details of statistical methods for each experiment were indicated in the respective figure legends. Results are expressed as means ± SD. For comparisons between groups, statistical analyses were performed using the Mann–Whitney test. *P*-values are presented as star marks in figures: ^*^P < 0.05, ^**^P < 0.01, ^***^P < 0.001. No statistical analysis was used to predetermine sample size and the suitability of statistical approaches.

## Results

### YTHDC2 Is Highly Expressed in Radiation-Resistant NPC Cells and Clinical Samples

**Figure 1:**
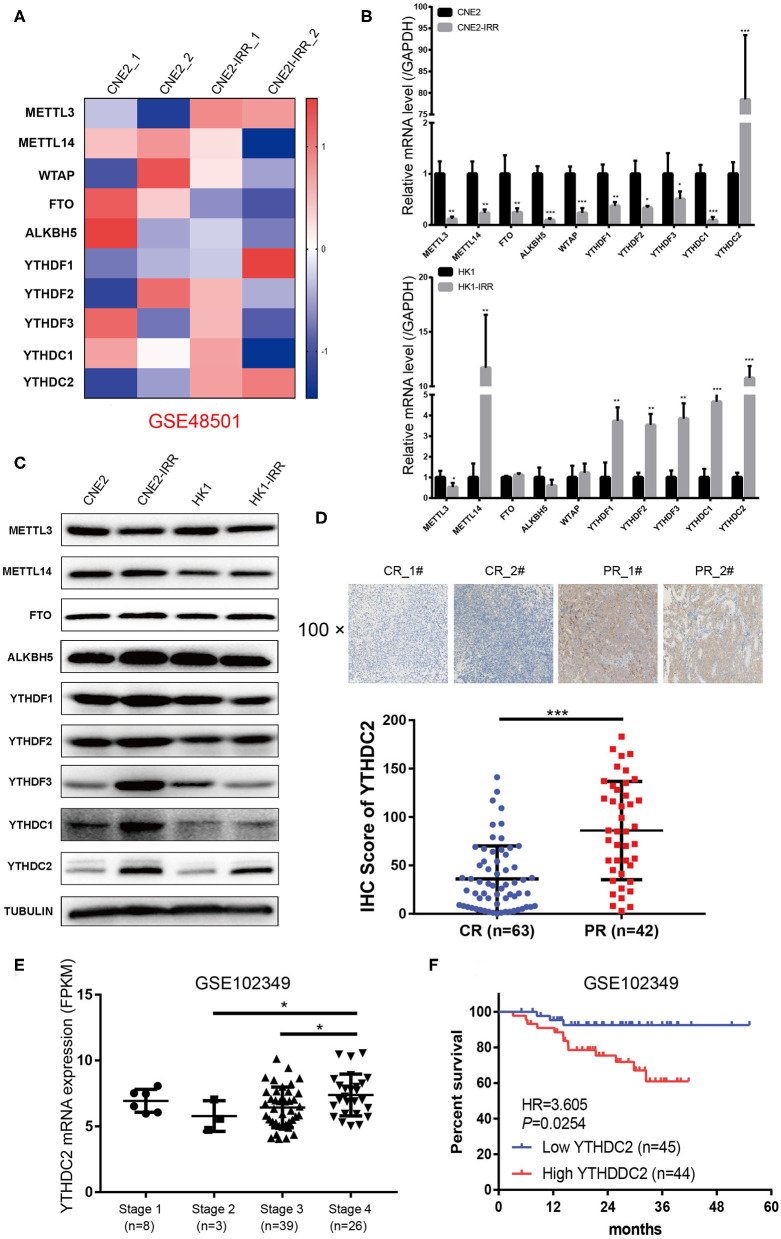
YTHDC2 is upregulated in radiation-resistance NPC cells. **(A)** Heat map showing the expression of m6A relevant genes in CNE2 and CEN2-IRR cells. The shades of red and blue represent increases and decreases in the expression of the corresponding genes, respectively. Data were generated in independent experiments (1 and 2). **(B,C)** mRNA **(B)** and protein **(C)** expression of the m6A relevant genes in CNE2, CNE2-IRR, HK1, and HK1-IRR cells. Data are presented as mean ± SD from *n* = 3. *P < 0.05, **P < 0.01, ***P < 0.001, student's *t* test. **(D)** H-Score of YTHDC2 in NPC sample with complete response and with partial response after radiotherapy. Up-panel showed representative images of YTHDC2 protein level examined in NPC CR and PR tissues using immunohistochemistry (shown in 100×magnifications). ***P < 0.001, student's *t* test. **(E)** scatter plots comparing levels of YTHDC2 mRNA in different clinical stage groups of NPC samples from GSE102349. *P < 0.05, Student's *t*-test. **(F)** Recurrence-free survival rate analysis base on YTHDC2 mRNA expression in NPC patients from GSE102349.

To examine the expression of the genes involved in m^6^A modification, an analysis of an NPC transcriptome-sequencing dataset (GSE48501 including radiosensitive cell line CNE2 and radioresistant cell line CNE2-IRR) from the GEO database was performed. Among all the genes, METTL3 and YTHDC2 were found to be expressed at remarkably higher levels in the CNE2-IRR cell line than in the parental cell line CNE2 (Figure 1A). Then we examined the level of YTHDC2 mRNA and protein in two pairs of radiosensitive and radioresistant NPC cell lines (CNE2 and CNE2-IRR, and HK1 and HK1-IRR, respectively) to further confirm this finding. As shown in Figures 1B,C, a significant elevation of the mRNA and protein levels of YTHDC2 was observed in CNE2-IRR and HK1-IRR, suggesting that YTHDC2 was upregulated in radioresistant NPC cells.

Next, we examined NPC tissues containing 105 patient specimens by immunohistochemistry (IHC) assay. We found that YTHDC2 expression was elevated in patient specimens of partial response (PR) compared to those of complete response (CR) (Figure 1D). Furthermore, higher protein expression of YTHDC2 was negatively correlated with the radiotherapy efficacy (Table 1). In addition, the highest expression of YTHDC2 was observed in clinical stage 4 of NPC patients (Figure 1E). Subsequently, we used GES102349 to analyze the association of YTHDC2 expression with Recurrence-free survival (RFS), and found that the low expression level of YTHDC2 mRNA was significantly correlated with better prognosis (Figure 1F).

**Table 1:** Clinical characteristics of 105 NPC patients according to YTHDC2 expression levels.

	**YTHDC2**				
**Feature**	**Low**	**High**	***n***	**χ^2^**	***P***
All cases	64	41	105		
**Gender**					
Male	44	21	65	3.257	0.099
Female	20	20	40		
**Age (years)**					
≤50	38	31	69	2.923	0.097
>50	26	10	36		
**TMN**					
Stage I + Stage II	40	21	61	1.306	0.312
Stage III + Stage IV	24	20	44		
**Tumor size (mm)**					
≤30	33	17	50	1.022	0.326
>30	31	24	55		
**Radiotherapy effect**					
Complete response	37	15	52	4.505	0.034^*^
Partial response	27	26	53		
**Pathological type**					
Undifferentiated non-keratinizing carcinoma	29	18	47	0.02	0.887
Differentiated non-keratinizing carcinoma	35	23	58		

*χ^2^ test was used to test the association between two categorical variables*.

* *Statistically significant*.

### Knocking Down of YTHDC2 Alleviates Radioresistance of NPC Cells

**Figure 2:**
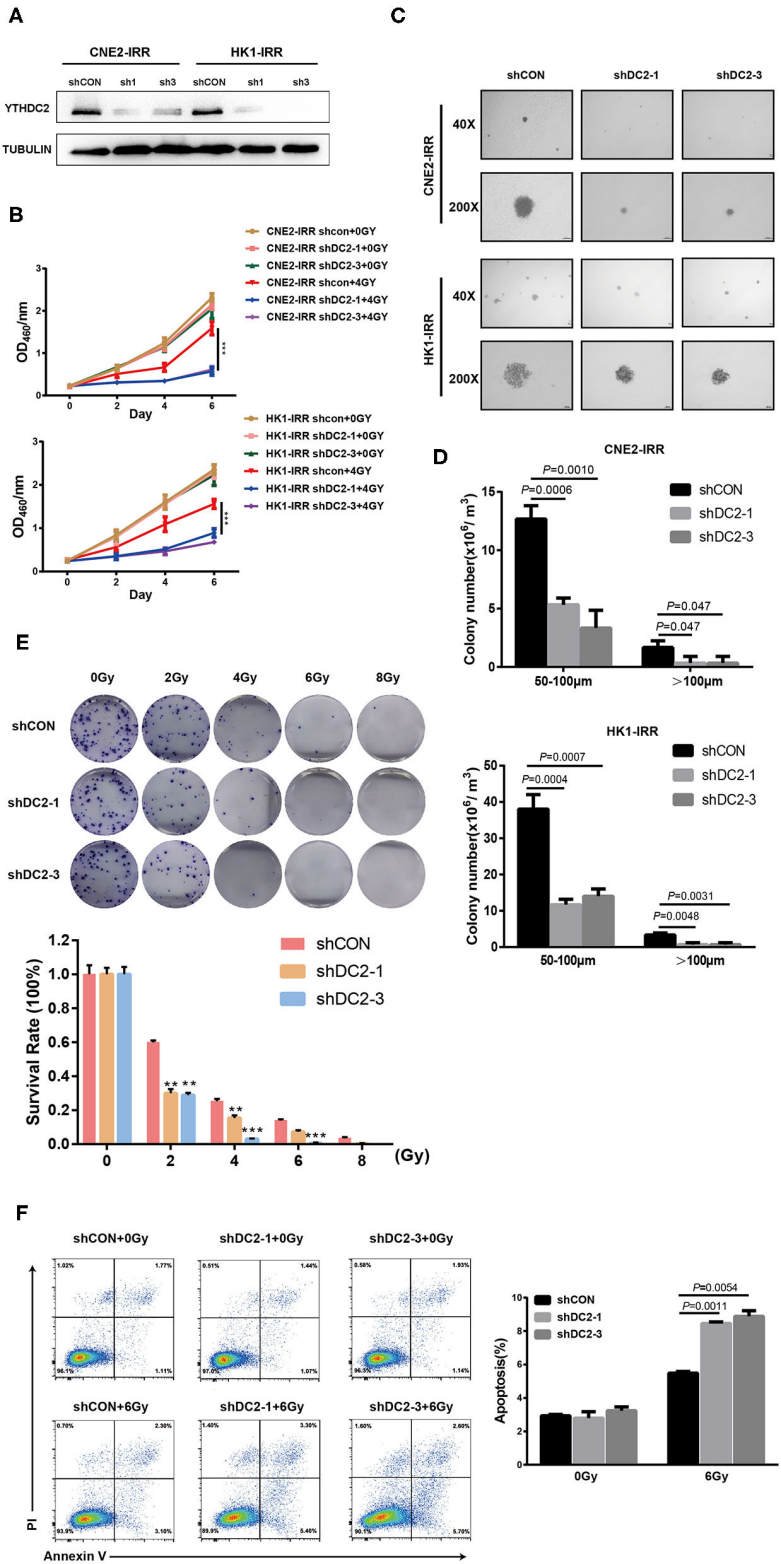
Loss of function of YTHDC2 enhances radiosensitivity of NPC cell *in vitro*. **(A)** Demonstration of shRNA efficiency in down-regulation of YTHDC2 in CNE2-IRR and HK1-IRR cells. **(B)** CCK8 assays of CNE2-IRR and HK1-IRR cells with knockdown of YTHDC 2 with or without irradiation treatment. Data are presented as mean ± SD from *n* = 3. ***P < 0.001, student's *t*-test. **(C)** Images from soft agar colony formation assay in CNE2-IRR and HK1-IRR cells with YTHDC2 knockdown after expose to radiation were shown in 2 different magnifications (40×and 200×). Data are presented as mean ± SD from *n* = 4. **(D)** Quantitative analyses of colony size (50–100 μm and >100 μm) in CNE2-IRR and HK1-IRR cells with YTHDC2 knocking down after exposure to radiation. **(E)** Survival rate in CNE2-IRR cells expose to 0, 2, 4, 6, 8 Gy radiation after YTHDC2 was knocked down. Data are presented as mean ± SD from *n* = 3. **P < 0.01, ***P < 0.001, student's *t*-test. **(F)** The apoptosis assay of CNE2-IRR with YTHDC2 knockdown after 0 or 6 Gy radiation by flow cytometric analysis (left) and the statistical analysis of the data (right). Data are presented as mean ± SD from *n* = 3, student's *t*-test.

An association between YTHDC2 expression and the radiotherapy effect in clinical samples and cell lines prompted us to explore the impact of YTHDC2 on radioresistance. We first knocked down the endogenous YTHDC2 by using lentiviral infection of two different short hairpin (sh)-RNAs in CNE2-IRR and HK1-IRR cells (Figure 2A). The YTHDC2 knockdown significantly suppressed NPC cells survival after exposure to irradiation (IR) (Figure 2B). In order to assess the effect of YTHDC2's knockdown on anchorage-independent growth in response to irradiation, we performed soft agar colony assays. After administering 6 Gy of irradiation as a single dose, knockdown of YTHDC2 obviously inhibited NPC cell proliferation and reduced colony numbers and size compared with the control (Figures 2C,D). In an IR dose-dependent assay, knocking down of YTHDC2 expression led to a significant reduction of colony growth at all the doses (Figure 2E). We also found that downregulating YTHDC2 led to increased apoptosis in the IR-treated radioresistant NPC cells by using flow cytometry (Figure 2F).

### Overexpression of YTHDC2 Attenuated the Irradiation Effect on Radiosensitive NPC Cells

**Figure 3:**
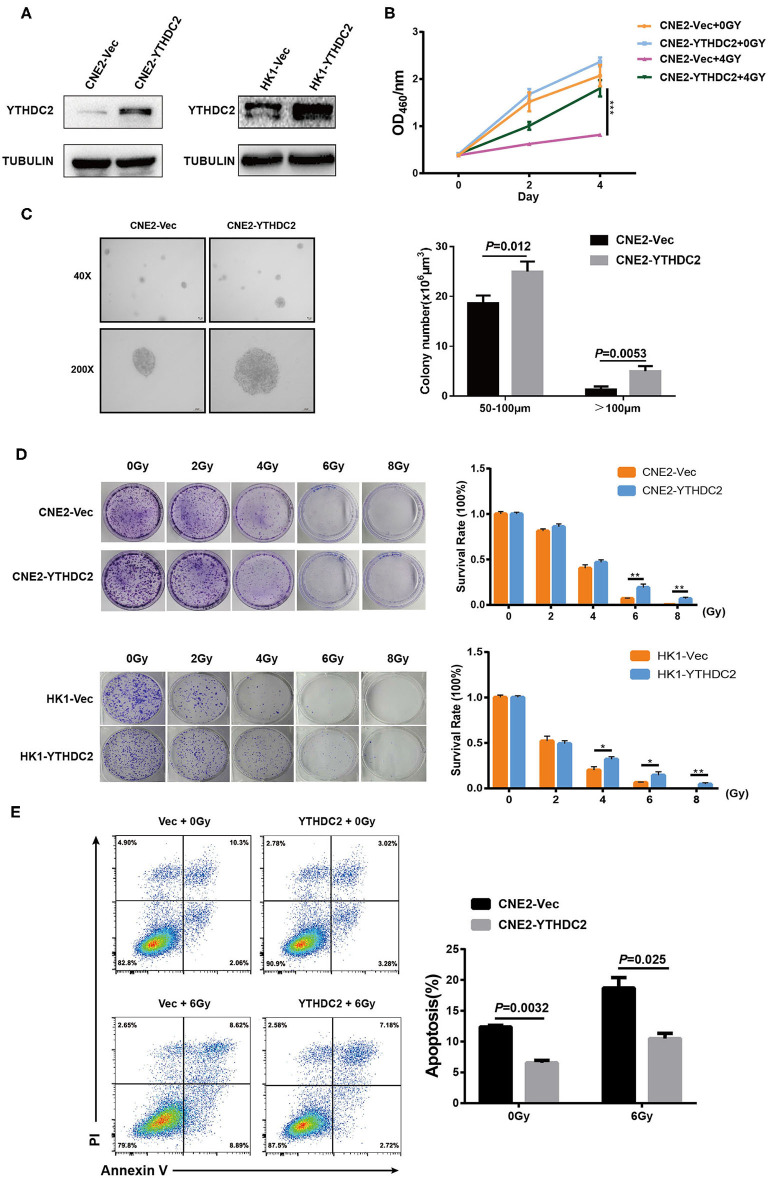
Overexpression of YTHDC2 attenuates radiosensitivity of NPC cell *in vitro*. **(A)** Demonstration of YTHDC2 over-expression in CNE2 and HK1 cells. **(B)** CCK8 assay indicated overexpression of YTHDC2 weaken radiotherapy effect in CNE2 cells. Data are presented as mean ± SD from *n* = 3. ***P < 0.001, student's *t* test. **(C)** Images of soft agar colony formation assay in CNE2 cells after expose to radiation were shown in 2 different magnifications (40×and 200×) (left) and statistical analysis of colony numbers (50–100 μm and >100 μm) (right). Data are presented as mean ± SD from *n* = 4. **(D)** The colonies (left) and survival rate (right) in CNE2 and HK1 cells expose to 0, 2, 4, 6, 8 Gy radiation with overexpression of YTHDC2. Data are presented as mean ± SD from *n* = 3. **P < 0.01, student's *t*-test. **(E)** The apoptosis assay of CNE2 with overexpression of YTHDC2 after 0 or 6 Gy radiation exposure by flow cytometric analysis (left) and data analysis (right). Data are presented as mean ± SD from *n* = 3, student's *t*-test. *P < 0.05, student's *t*-test.

Next, we examined whether the enforced expression of YTHDC2 impacted the response of cancer cells to irradiation. YTHDC2 was overexpressed in the radiosensitive NPC cell line CNE2 and HK1 (Figure 3A). As shown in Figure 3B, overexpression of YTHDC2 promoted cell survival after exposure to 4 Gy irradiation. The enhanced cell proliferation and anchorage-independent growth could be observed in CNE2-YTHDC2 cells compared to the control (Figure 3C). When the cells were treated with different doses (0, 2, 4, 6, 8 Gy), irradiation resistance was particularly conferred to CNE2-IRR at 6 and 8 Gy and to HK1-IRR at 4, 6, and 8 Gy (Figure 3D). Finally, we found that YTHDC2 overexpression dramatically decreased IR-induced apoptosis in CNE2 (Figure 3E). Collectively, high expression of YTHDC2 promoted radiotherapy resistance of NPC.

### YTHDC2 Regulates Cellular Response to Irradiation via PI3K-AKT/S6 Pathway

**Figure 4:**
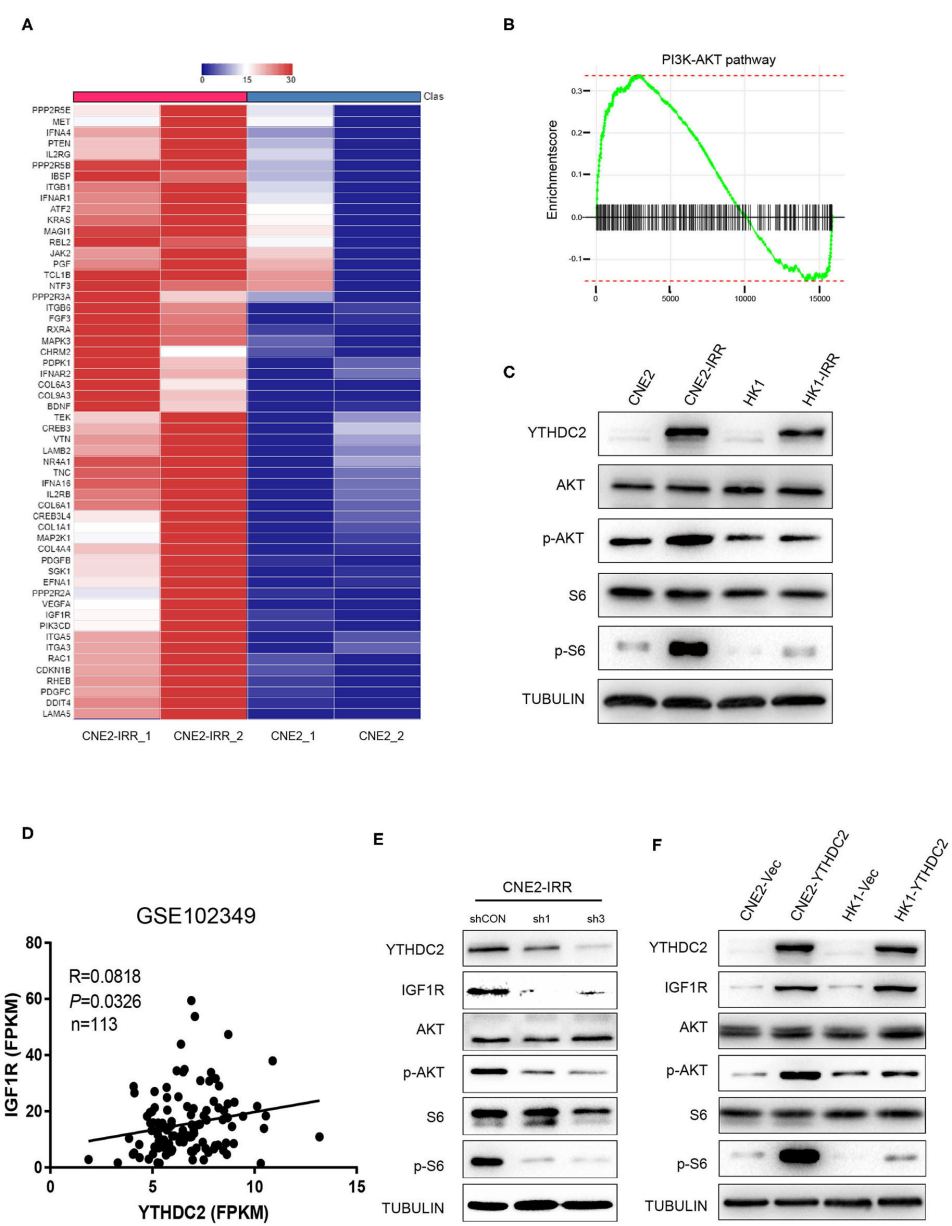
IGF1R is a key downstream gene of YTHDC2. **(A)** Fifty-six genes related to the PI3K-AKT/S6 signaling pathway were found to be upregulated in the GSE48501 dataset, among which IGF1R is the key downstream target gene of YTHDC2 by using m6A prediction database (m6avar.renlab.org) **(B)** PI3K-AKT signaling was activated in radiation-resistance NPC cells. **(C)** Western blot analyses of PI3K-AKT/S6 signaling activation in radiation resistant cells. **(D)** A positive correlation between the expressions of YTHDC2 and IGF1R in NPC tissues was found by analyzing GSE102349 dataset. **(E)** Knockdown of YTHDC2 in CNE2-IRR cells downregulated IGF1R protein expression and inactivated PI3K-AKT/S6 signaling. **(F)** Overexpression of YTHDC2 in CNE2 and HK1 cells upregulated IGF1R protein expression and activated PI3k-AKT signaling. All experiments were repeated twice.

In order to investigate the potential mechanism underlying radioresistance mediated by YTHDC2 in NPC cells, we searched the literature to analyze potential signaling pathways involved in radiation response, and we found that PI3K-AKT actively participated in radioresistance of cancers (37–40). To validate this, we conducted data mining of a public RNA-Seq dataset GSE48501 composed of RNA-Seq profiles of CNE2 and CNE2IRR cell lines. Fifty-six genes related to the PI3K-AKT/S6 signaling pathway were found to be upregulated in the GSE48501 dataset (Figure 4A). The PI3K-AKT/S6 signaling pathway in CNE2-IRR cells was examined and found to be significantly activated (Figures 4B,C). Since YTHDC2 is reported to function as a m^6^A reader by binding to specific sites of RNAs, we searched the m6AVar and RMbase databases (41, 42) and identified mRNAs of 32 candidate genes encompassing potential YTHDC2 binding site (Supplementary Table 2). We found that insulin-like growth factor 1 receptor (IGF1R) might be a putative gene mediated by YTHDC2. In addition, the analysis of GSE102349 dataset identified a significant positive correlation between YTHDC2 and IGF1R expression (Figure 4D).

To confirm the regulatory role of YTHDC2 for IGFR1, we knocked down YTHDC2 expression in CNE2-IRR cells or overexpressed YTHDC2 in CNE2 and HK1 cells, and we found that depletion of YTHDC2 downregulated protein expression of IGF1R and inhibited downstream PI3K-AKT/S6 signaling (Figure 4E), while YTHDC2 overexpression increased the protein level of IGF1R and activated PI3K-AKT/S6 signaling (Figure 4F).

### Promoter Hypo-Methylation Increases Level of YTHDC2

**Figure 5:**
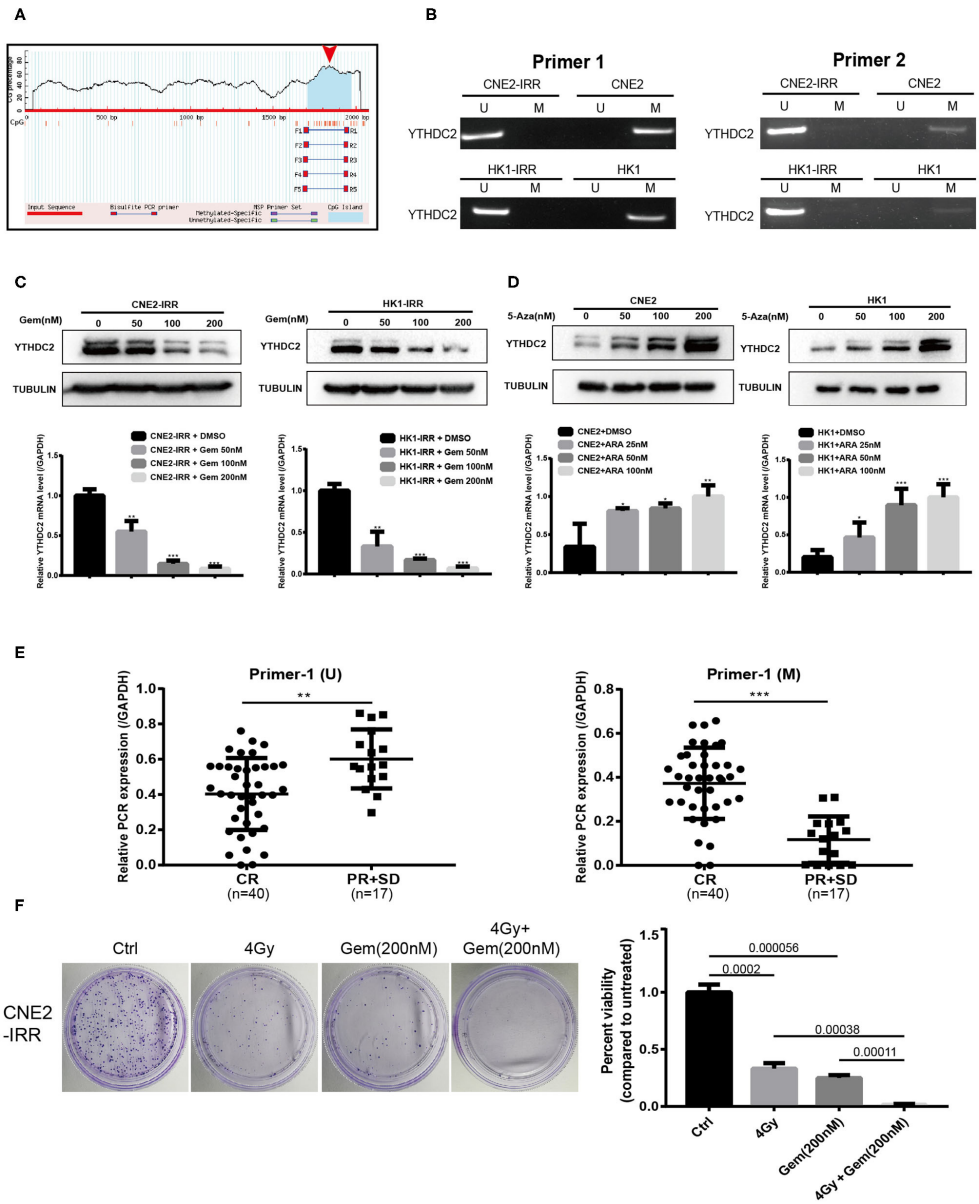
Promoter methylation regulates gene expression level of YTHDC2. **(A)** Schematic representation of the CpG distribution in promoter region of the YTHDC2 gene from the CpG Island Searcher (https://www.urogene.org/cgi-bin/methprimer/methprimer.cgi). **(B)** Methylation-specific PCR (MSP) analysis of YTHDC2 promoter methylation status in two pairs of cell lines using 2 serials of primers. **(C)** YTHDC2 expression in CNE2-IRR and HK1-IRR cells treated with gemcitabine with different concentrations for 48 hr and subjected to immunoblotting and qPCR assay. Data are presented as mean ± SD from *n* = 3. *P < 0.05, **P < 0.01, ***P < 0.001, student's *t*-test. **(D)** CNE2 and HK1 cells treated with 5-aza-2'-deoxycytidine with different concentrations for 48 hr and subjected to immunoblotting and qPCR assay of YTHDC2 expression. Data are presented as mean ± SD from *n* = 3. *P < 0.05, **P < 0.01, ***P < 0.001, student's *t* test. **(E)** MSP analysis of YTHDC2 promoter methylation in paraffin-embeded sections of radiosensitivity and radioresistance NPC tissues. Data are presented as mean ± SD from *n* = 3. **P < 0.01, ***P < 0.001, student's *t*-test. **(F)** CNE2-IRR cells were treated with radiotherapy, gemcitabine, radiotherapy plus gemcitabine, respectively. The same number of cells were seeded and the colonies formed were counted 14 days later.

Next, we investigated the mechanism underlying the higher expression of YTHDC2 in resistant NPC cells. By analyzing the gene promoter methylation level of HNSC from TCGA, we found that the promoter methylation level of YTHDC2 was lower in primary tumors than that in normal tissues (Supplementary Figure 1A), and subgroup analysis indicated that the lowest promoter methylation was observed in a group of pathological grade 4 tumors (Supplementary Figure 1B). On the basis of this analysis, we speculated that expression of YTHDC2 might be regulated by promoter methylation. In order to validate this, we utilized an online tool to analyze the G/C content and methylation status in the promoter of YTHDC2 (https://www.urogene.org/methprimer/) and found that G/C rich regions were distributed in the transcriptional starting site of YTHDC2 gene, and a methylated CpG island (CGI) was located at the promoter of YTHDC2 (Figure 5A). Next, we performed MSP to examine the methylation of YTHDC2 promoter in parental cells and radioresistant cells. The results showed that the methylation levels of YTHDC2 in the resistant cells was significantly lower than that of control cells (Figure 5B). Indeed, treatment with gemcitabine, a pharmacological inhibitor of DNA demethylation, dramatically decreased the protein and mRNA levels of YTHDC2 in both CNE2-IRR and HK1-IRR cells (Figure 5C). In contrast, 5-azacytidine, a DNA methyltransferase inhibitor, apparently enhanced the protein and mRNA levels of YTHDC2 in both CNE2 and HK1 cells (Figure 5D), which implied that the lower methylation level in the YTHDC2 promoter in radioresistant NPC cells was responsible for high levels of YTHDC2. PCR quantitative analysis of MSP showed that the promoter methylation of YTHDC2 in NPC tissues with radioresistance was lower than that of NPC tissues without radioresistance (Figure 5E). The combination of radiotherapy and gemcitabine showed better therapeutic effects than radiotherapy or gemcitabine alone (Figure 5F).

### YTHDC2 Increases IGF1R Expression via Promoting Translation Efficiency of IGF1R

**Figure 6:**
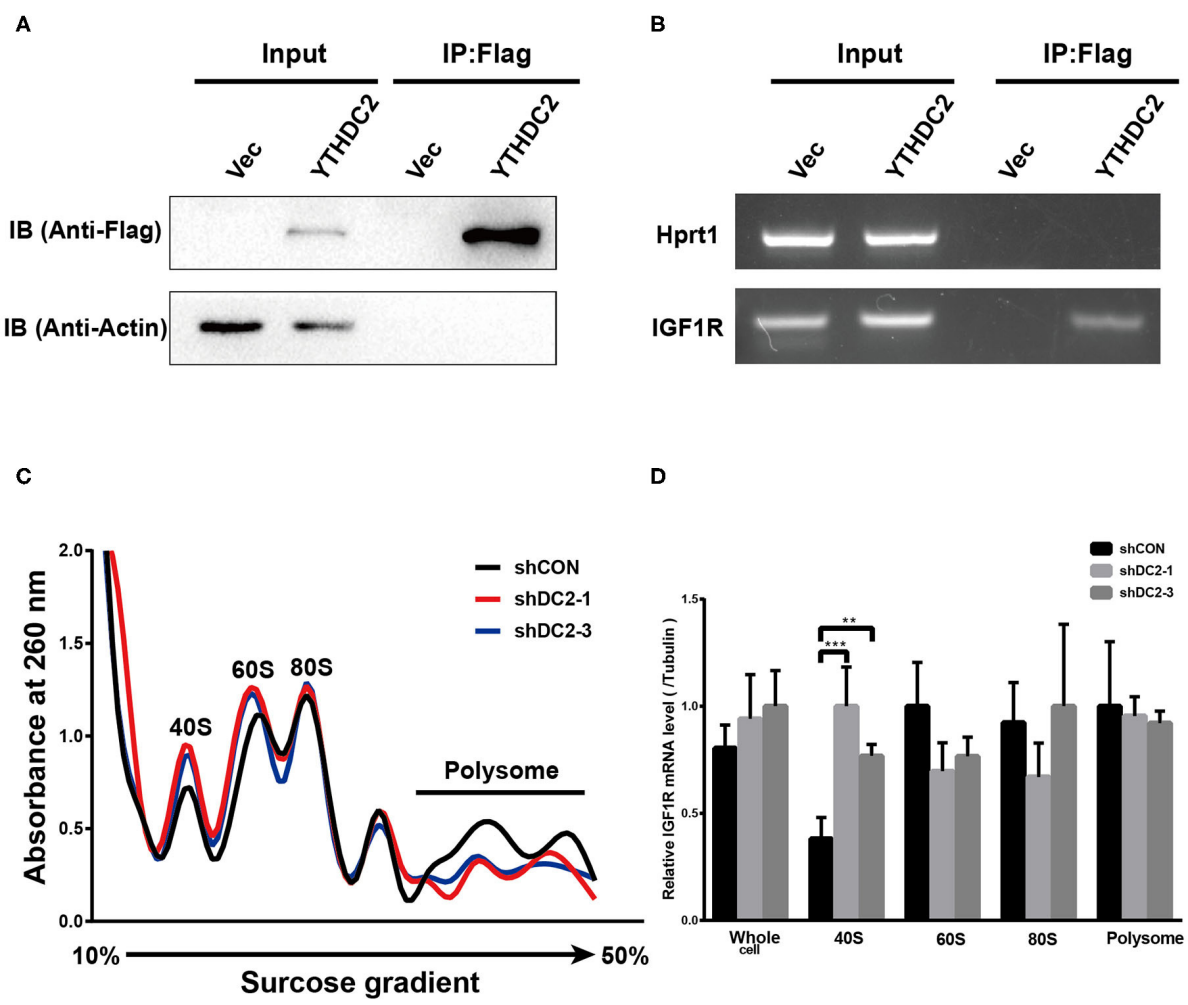
YTHDC2 regulates translation of IGF1R. **(A)** Verification of efficiency of FLAG-immunoprecipitation by western blot. **(B)** RNA immunoprecipitation of FLAG-YTHDC2 interacted with IGF1R *in vivo* in CNE2 cells. Protein–RNA complexes immunoprecipitated by anti-FLAG or IgG were determined by RT-PCR using specific primers for IGF1R or Hprt1 (negative control). **(C)** Cell lysate derived from shCON, shYTHDC2-1, and shYTHDC2-3 of CNE2-IRR cells were loaded on a linear gradient of 15–40% sucrose and centrifuged at 38,000 rpm at 4°C for 4 hr. After centrifugation, samples were then fractionated into 24 fractions (0.5 mL per fraction), respectively and were monitored at 260 nm. **(D)** The amount of IGF1R mRNA in 40S, 60S, 80S, polysome fractionated RNA and total RNA derived from shCON, shYTHDC2-1, and shYTHDC2-3 of CNE2-IRR cells were quantified by quantitative RT-PCR. Data are presented as mean ± SD from *n* = 3. **P < 0.001; ***P < 0.001, student's *t*-test.

YTHDC2 is a member of the DEAH (Asp-Glu-Ala-His) subfamily of proteins, part of the DEAD (Asp-Glu-Ala-Asp) box family of RNA helicases. It was reported that several DEAH/D box RNA helicases promote translation initiation of mRNA (43, 44). In addition, recent findings showed that the elongation-promoting effect of CDS m^6^A modification requires the RNA helicase-containing m^6^A reader YTHDC2 (25). Thus, we examined if YTHDC2 could modulate IGF1R expression by affecting translation initiation. We performed an RNA immunoprecipitation (IP) assay to detect the potential association between exogenous YTHDC2 protein and IGF1R mRNA in CNE2 cells. Firstly, western blot was performed to validate the expression level of Flag-YTHDC2 and the efficiency of RNA-immunoprecipitation (Figure 6A). The RNA IP assay showed that IGF1R mRNA was significantly enriched in the RNAs immunoprecipitated with the anti-Flag antibody in the Flag-YTHDC2 overexpressing CNE2 cells, compared with the control vector-infected CNE2 cells, indicating that the YTHDC2 protein bound to IGF1R mRNA (Figure 6B). Furthermore, polysome profiling indicated that knockdown of YTHDC2 resulted in the accumulation of IGF1R mRNA in 40S initiation complexes (Figures 6C,D). In addition, knockdown of YTHDC2 has little effect on transcription and stability of IGF1R mRNA (Supplementary Figures 2A,B). Taken together, YTHDC2 may enhance the translation efficiency of IGF1R mRNA.

### Knockdown of YTHDC2 Sensitizes NPC to Radiotherapy *in vivo*

**Figure 7:**
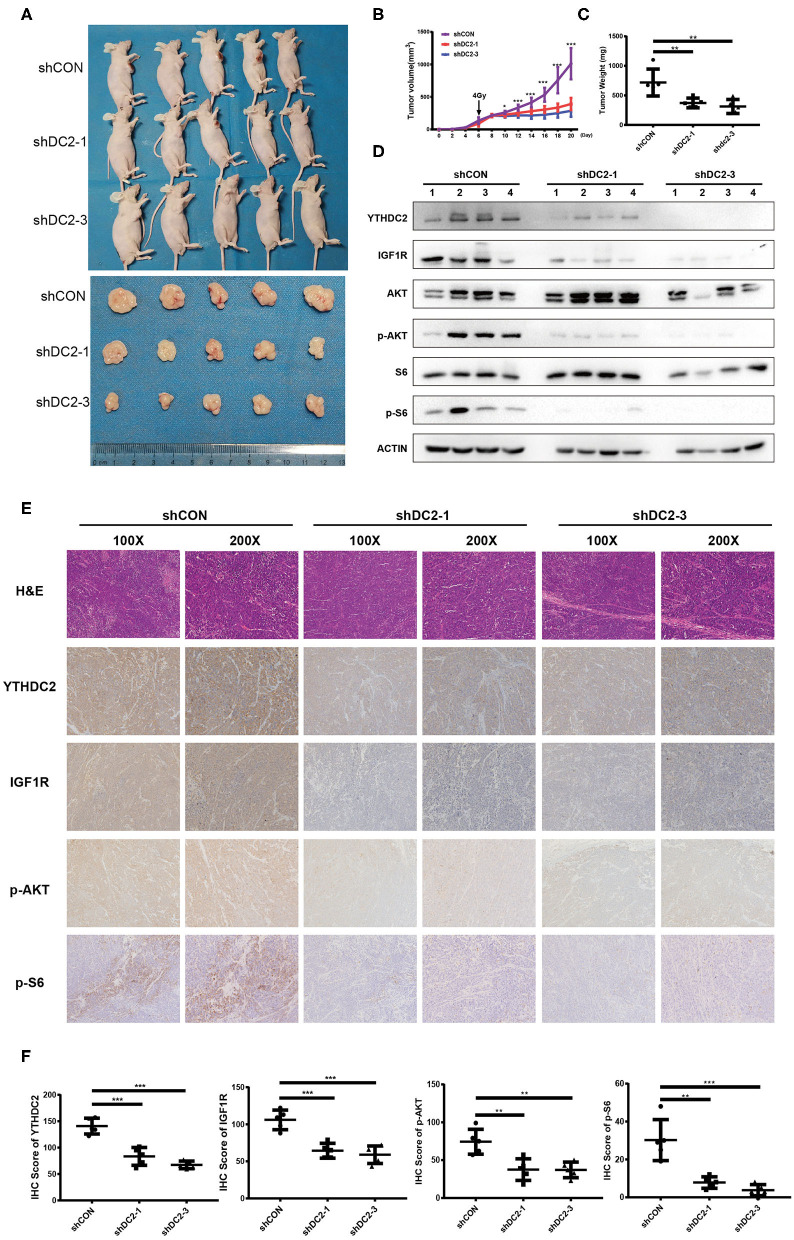
Knockdown of YTHDC2 enhances radiotherapy effect *in vivo*. **(A)** Knockdown of YTHDC2 significantly enhanced radiotherapy effect (*n* = 5). One dose of irradiation was given at the day 6 when tumor volumes reached around 100 mm^3^. Photos were taken from tumor samples collected at the end of experiment (day 21). **(B)** The growth curve of tumors from CEN2-IRR cells transduced with viral vectors expressing shRNA-1, shRNA-3, or control sequence (*n* = 5). **(C)** Tumors weight measured at the end of experiment (day 21) (*n* = 5). **(D)** The YTHDC2 expression and inhibition of PI3K-AKT/S6 pathway in tumors was determined by western blot. **(E)** Representative images of immunohistostaining for examining the expression of YTHDC2, IGF1R, p-AKT, and p-S6 in xenograft tissues. Images were shown in 2 different magnifications (100×and 200×). **(F)** Quantitative analysis of YTHDC2, IGF1R, p-AKT, and p-S6 expression in the xenograft tumors. All experiments were repeated twice. *P < 0.05, **P < 0.01, ***P < 0.001, student's *t*-test.

To confirm our observations of the effect of YTHDC2 on radiosensitivity, an NPC xenograft mouse model was established by using a subcutaneous injection of CNE2-IRR cells infected with YTHDC2 shCON, shYTHDC2-1, and shYTHDC2-3 lentivirus, respectively. Firstly, we found that knockdown of YTHDC2 had little effect on tumor growth without radiotherapy (Supplementary Figures 3A–C). However, the mice with YTHDC2 shRNA transduced cells had slow growth, smaller tumor volumes, and less weight than the control tumor after irradiation treatment (Figures 7A–C). Western blot and IHC assays of xenografts confirmed that depletion of YTHDC2 downregulated IGF1R expression and inactivated PI3K-AKT/S6 signaling (Figures 7D–F). Thus, knockdown of YTHDC2 promotes the radiotherapy effect of NPC *in vivo*.

## Discussion

Radiation resistance remains a major clinical problem, resulting in a poor outcome for patients with cancer. Several mechanisms appeared to be involved in intrinsic or acquired resistance to radiotherapy, e.g., altered DNA repair ability (45, 46), activated survival signaling (47, 48), decreased reactive oxygen species accumulation (49), and enhanced percent of cancer stem cells (50, 51). Recent studies showed that m^6^A methylation of RNAs plays important roles in various cancers. However, it is unclear whether functional proteins participating in m^6^A modification are involved in regulating radiation resistance. Herein, we found the aberrantly high expression of YTHDC2, an m^6^A reader protein, in radioresistant NPC cells, which might be due to hypomethylation of the YTHDC2 promoter. YTHDC2 could activate the PI3K-AKT/S6 pathway by regulating the translation of IGF1R mRNA. In consistence of our conclusion, several studies revealed that the PI3K-AKT pathway played an important role in a tumor's radiation response (52–55).

It is widely accepted that the status of promoter methylation is associated with inhibition of gene expression. In the mammalian genome, there are two DNA methylation patterns (CpG dinucleotides and short DNA stretches called CGIs). In fact, all CpG dinucleotides are methylated with the general exception of CGIs, and the high methylation of CGIs is generally associated with the inhibition of promoter activity (56). Moreover, alteration of the methylation level in the promoter might be an adaptive response to long exposure of radiation (57–59). In the present study, a 212 bp sequence was identified as CGI in the YTHDC2 promoter predicted by MethPrimer. Furthermore, there was a dramatically decreased expression of YTHDC2 in radioresistant cells with treatment of gemcitabine. In contrast, 5-azacytidine can promote the accumulation of YTHDC2. Therefore, the low methylation level of the promoter may lead to the higher expression of YTHDC2 in radioresistant NPC cells. Based on our observations, gemcitabine may sensitize NPC cells to radiation by decreasing the expression of YTHDC2, which presents a possible explanation of the efficacy of gemcitabine treatment in recurrent or metastatic NPC (60, 61), and implies that YTHDC2 might be a promising therapeutic target for recurrent NPC.

In our study, we found that YTHDC2 regulated the protein expression of IGF1R by affecting translation IGF1R mRNA, which is consistent with the previous studies. IGF1R is a transmembrane receptor activated by a hormone called insulin-like growth factor 1 (IGF-1) and by a related hormone called IGF-2. Ligand binding activates the receptor kinase (such as IGF1R), subsequently leading to increasing activation of downstream pathways (such as the PI3K-AKT/PKB pathway and Ras-MAPK pathway). The resultant activation of the MAPK pathway will lead to cell proliferation, whereas activation of the PI3K pathway inhibits apoptosis and stimulates protein synthesis. Hyperactive PI3K/AKT signaling may account for the increased radiation resistance of cancer cells. It has been suggested that AKT is the key molecule in PI3K-mediated radioresistance by promotion of the repair of IR-induced DNA double-strand breaks, maintenance of the stemness of cancer cells, and inhibition of IR-induced autophagy (52, 54). In our study, IGF1R was upregulated by overexpression of YTHDC2, and then it promoted phosphorylation of AKT and S6, which are responsible for increasing resistance to radiation *in vivo* and *in vitro*.

**Figure 8:**
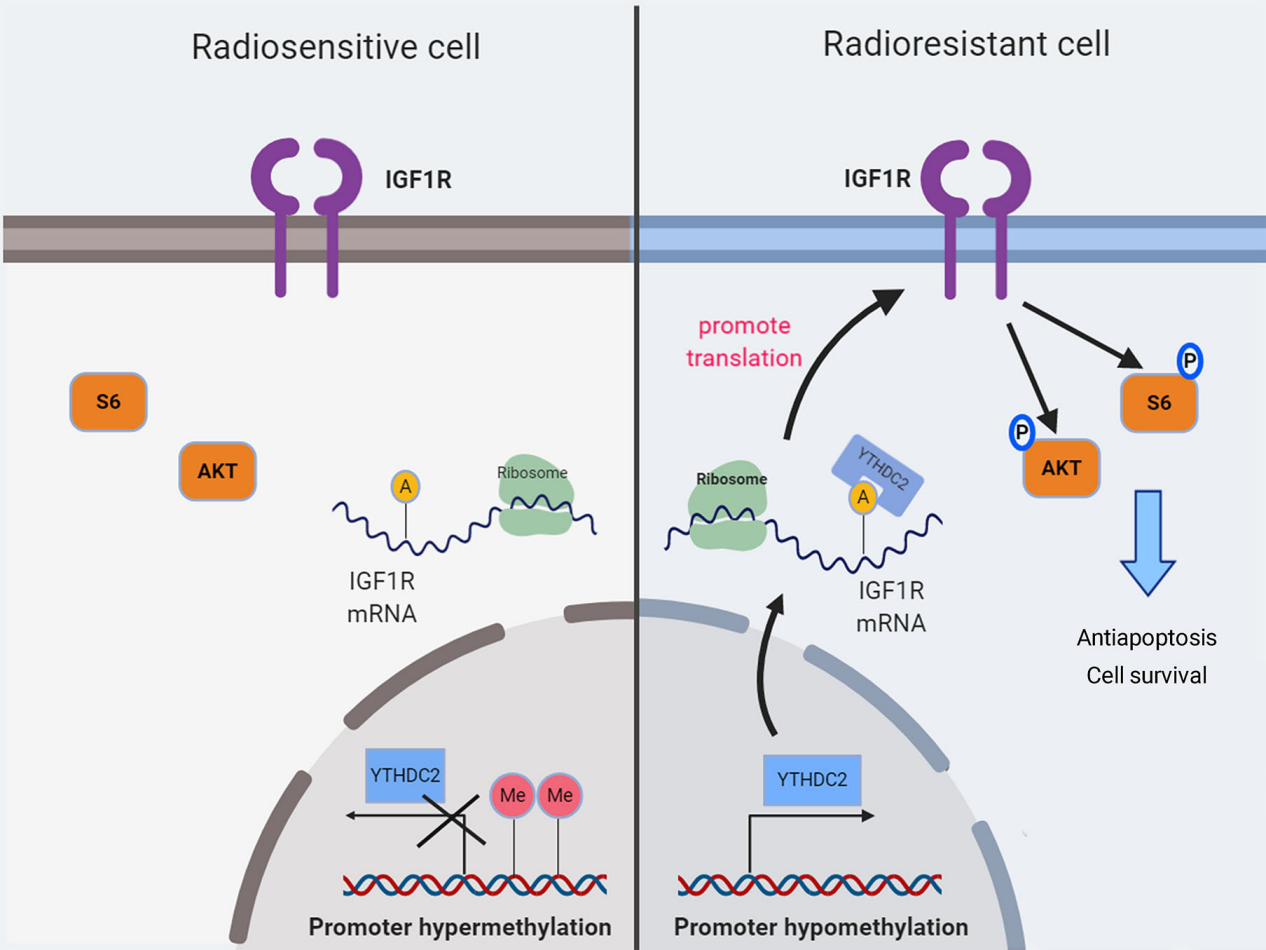
A schematic diagram showing YTHDC2 upregulated expression of IGF1R attenuate radiotherapy response via mediated RNA translation.

In summary, our findings demonstrated for the first time that the high expression of the m^6^a reader YTHDC2 in NPC cells promotes the translation efficiency of IGF1R mRNA, resulting in resistance to irradiation (Figure 8).

## Data Availability Statement

The raw data supporting the conclusions of this article will be made available by the authors, without undue reservation.

## Ethics Statement

The human study was approved by the Institute Research Medical Ethics Committee of Central South University. All samples were deidentified and all patients signed informed consents.

## Author Contributions

J-JH, Y-MD, QZ, and L-QS designed the study. J-JH, Y-MD, and ZL performed the experiments. J-JH, QF, and Y-MD performed the animal experiments. Z-XR, JG, YM, Y-DG, X-XR, Y-YZ, and L-YL performed the immunohistochemistry. J-JH, Y-MD, and MZ completed the statistics and interpreted the data. Y-ZD provided the critical suggestions. All authors contributed to the article and approved the submitted version.

## Conflict of Interest

The authors declare that the research was conducted in the absence of any commercial or financial relationships that could be construed as a potential conflict of interest.

## Supplementary Material

Supplementary Figure 1 Promoter methylation regulates gene expression level of YTHDC2 in HNSC. **(A)** Analysis of the promoter methylation level of primary tumor (HNSC) and normal tissue (Data from TCGA). **(B)** Different levels of the promoter methylation of YTHDC2 gene in different tumor differentiation grades by using TCGA data-analysis web (http://ualcan.path.uab.edu/index.html).

Supplementary Figure 2 Knockdown of YTHDC2 hardly affects transcription and stability of IGR1R mRNA. **(A)** YTHDC2 Knockdown efficiency in CNE2-IRR cell lines (left), and transcription of IGF1R mRNA (right). **(B)** The mRNA stability of IGF1R in CNE2-IRR cells transduced with depleted expression of shCON or (shDC2-1 or shDC2-3). ^*^^*^*P* < 0.01, student's *t* test.

Supplementary Figure 3 Knockdown of YTHDC2 exerts little influence on tumor growth *in vivo*. **(A)** Knockdown of YTHDC2 barely affects tumor growth (*n* = 4). **(B)** The growth curve of the tumor (*n* = 4). **(C)** Tumor weight (*n* = 4).

Supplementary Table 1 Primers used for realtime-PCR.

Supplementary Table 2 Identification of 32 candidate genes encompassing potential YTHDC2 binding site from m6AVar and RMbase databases.

Supplementary Table 3 Primers for Methylation-Specific PCR of YTHDC2 Promoter.
