# Supplementary figures and images for: m6A Reader YTHDC2 Promotes Radiotherapy Resistance of Nasopharyngeal Carcinoma via Activating IGF1R/AKT/S6 Signaling Axis
Source: Front Oncol. 2020 Jul 31;10:1166. doi: 10.3389/fonc.2020.01166 (PMC7411471; doi:10.3389/fonc.2020.01166)

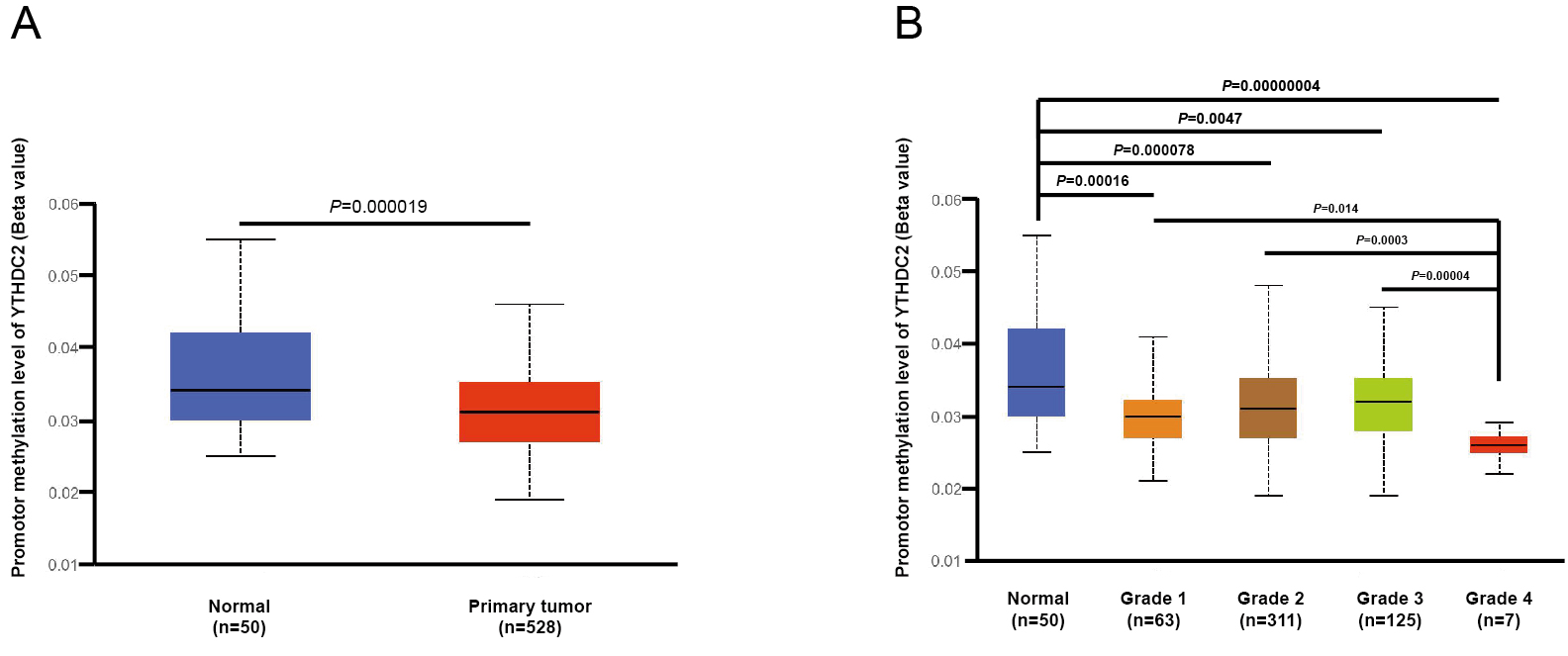

Supplement: Supplementary Figure 1 — Promoter methylation regulates gene expression level of YTHDC2 in HNSC. (A) Analysis of the promoter methylation level of primary tumor (HNSC) and normal tissue (Data from TCGA). (B) Different levels of the promoter methylation of YTHDC2 gene in different tumor differentiation grades by using TCGA data-analysis web (http://ualcan.path.uab.edu/index.html). [file Image_1.JPEG]

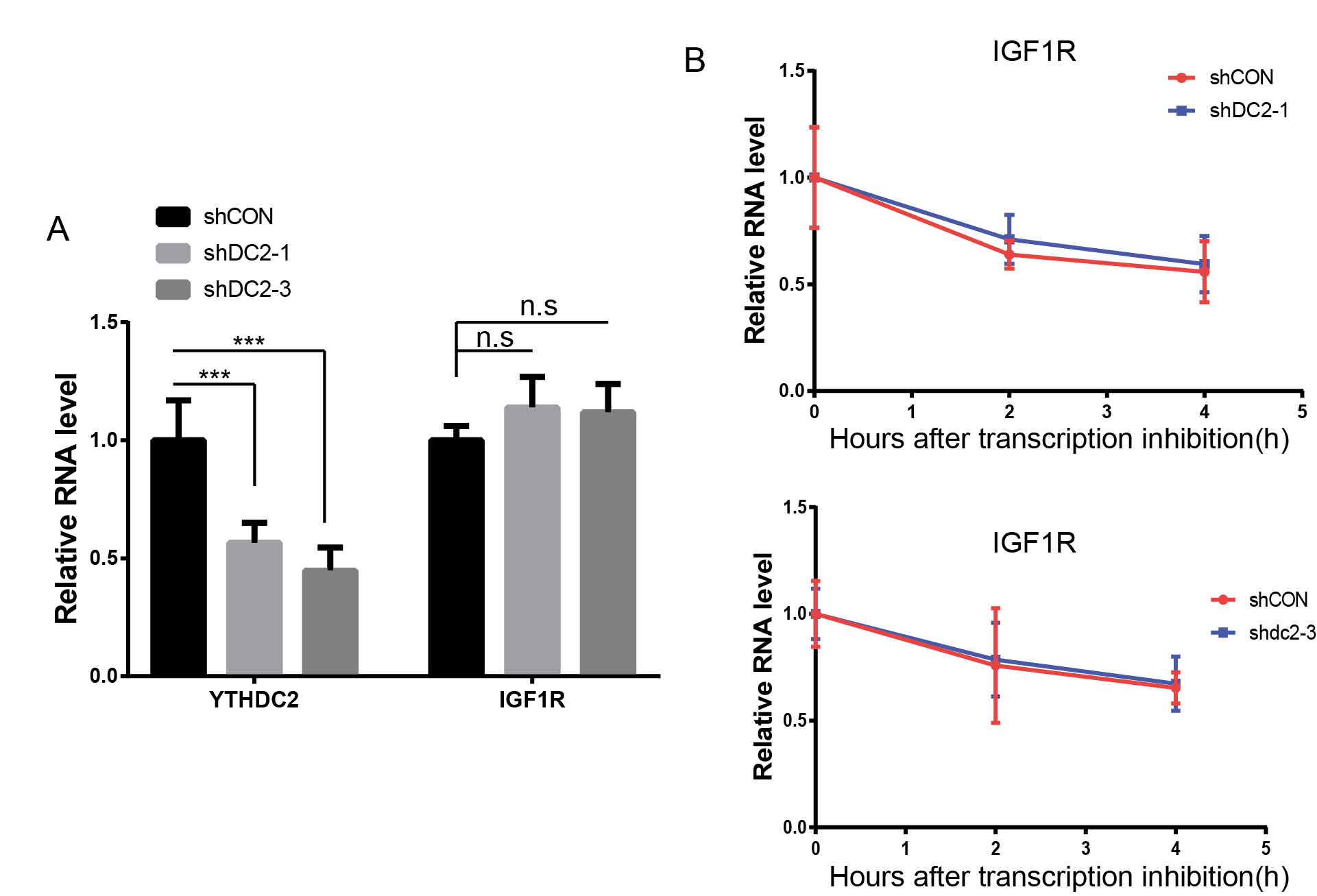

Supplement: Supplementary Figure 2 — Knockdown of YTHDC2 hardly affects transcription and stability of IGR1R mRNA. (A) YTHDC2 Knockdown efficiency in CNE2-IRR cell lines (left), and transcription of IGF1R mRNA (right). (B) The mRNA stability of IGF1R in CNE2-IRR cells transduced with depleted expression of shCON or (shDC2-1 or shDC2-3). **P < 0.01, student's t test. [file Image_2.JPEG]

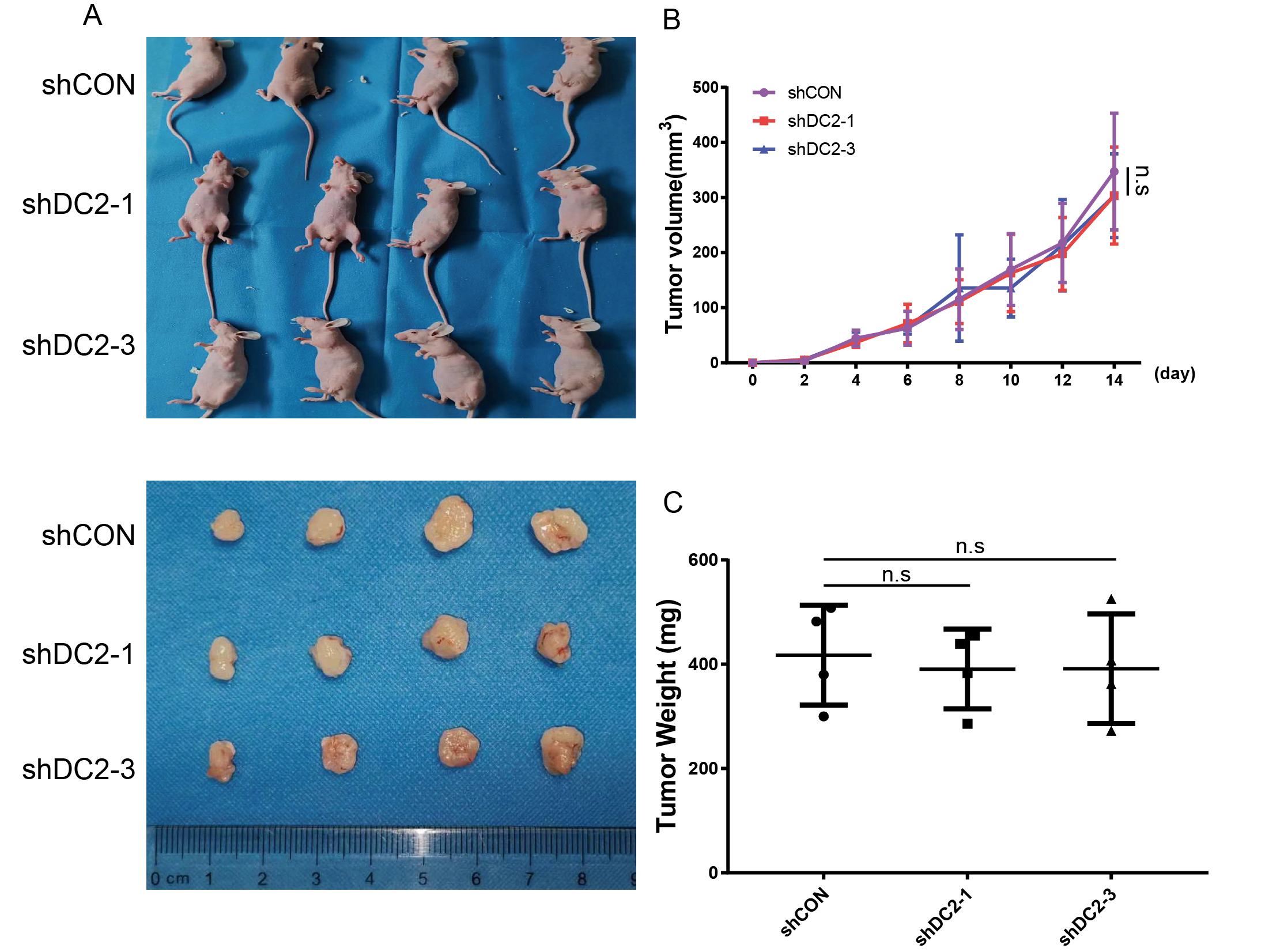

Supplement: Supplementary Figure 3 — Knockdown of YTHDC2 exerts little influence on tumor growth in vivo. (A) Knockdown of YTHDC2 barely affects tumor growth (n = 4). (B) The growth curve of the tumor (n = 4). (C) Tumor weight (n = 4). [file Image_3.JPEG]
